# Increased correction of lateral centre edge angle after periacetabular osteotomy is associated with a reduction in hip flexion and internal rotation: A three‐dimensional computed tomography simulation study

**DOI:** 10.1002/jeo2.70523

**Published:** 2025-11-28

**Authors:** Sufian S. Ahmad, Justus Stamp, Gaia Guidici, Quentin Karisch, Henning Windhagen, Marco Haertlé

**Affiliations:** ^1^ Department of Orthopaedic Surgery Hannover Medical School Hannover Germany

**Keywords:** CT simulation, hip flexion, hip dysplasia, internal rotation, periacetabular osteotomy, range of motion

## Abstract

**Purpose:**

Periacetabular osteotomy (PAO) is an established treatment for hip dysplasia, known to affect the hip range of motion (ROM). However, the quantitative relationship between PAO‐induced increase in lateral centre‐edge angle (LCEA) and postoperative ROM remains undefined. This study aimed to determine the precise influence of increased LCEA on hip ROM following PAO.

**Methods:**

Fifty computed tomography (CT)‐based PAO simulations were performed on dysplastic hips, with corrections to LCEA values of 25° and 35°. Hip ROM was assessed in multiple planes using collision‐based simulation endpoints. Linear regression and subgroup analyses evaluated the relationship between ΔLCEA and ROM changes.

**Results:**

Increased lateral acetabular coverage, as measured by the change in LCEA, showed significant negative correlations with hip flexion and internal rotation (both *p* < 0.001). Linear regression analysis quantified these relationships, indicating that for every 1‐degree increase in LCEA, internal rotation decreased by 1.003° (confidence interval [CI]: 0.7–1.3, *p* = 0.016) and flexion decreased by 1.36° (CI: 0.7–2.0, *p* < 0.001). Subgroup analysis further revealed that patients with lower preoperative ROM experienced more pronounced postoperative reductions in ROM. Notably, hips with < 20° preoperative internal rotation were at risk of fully losing internal rotation and having limited flexion if corrected to an LCEA of 35°.

**Conclusion:**

Greater correction of LCEA after PAO is associated with a measurable reduction in hip flexion and internal rotation. This study quantified these changes, finding a practical rule of thumb: for every 1‐degree increase in LCEA, internal rotation decreases by approximately 1.003°, and hip flexion decreases by about 1.36°. Notably, hips with preoperative internal rotation less than 20° were identified as high‐risk, as these hips were prone to fully losing internal rotation and experiencing limited flexion postoperatively. On the other hand, high internal rotation of >40° prior to PAO may be considered protective. To ensure residual ROM in hips at risk, the findings suggest accepting a lower target LCEA correction of 25°, on the lower end of the normal range.

**Level of Evidence:**

Level III.

**Abbreviations**
CT: computed tomography
IR: internal rotation
LCEA: lateral centre‐edge angle
PAO: periacetabular osteotomy
ROM: range of motion

## BACKGROUND

Periacetabular osteotomy (PAO) has gained widespread popularity as the gold‐standard procedure for the improvement of femoral head coverage in patients with developmental dysplasia of the hip (DDH) [2, 9, 15, 17]. Since its initial description, extensive research has focused on the diverse phenotypes of hip dysplasia, leading to an expansion of indications for PAO. Consequently, PAO has become the workhorse procedure for addressing a wide range of acetabular pathomorphologies [4, 5, 7, 11, 19, 20, 22, 24]. However, little is known about the target correction in PAO surgery for optimum long‐term outcome. Key elements of target correction were based on avoiding retroversion of the fragment while maintaining anterior wall coverage [18, 26, 28].

In the normal population, the lateral coverage, commonly measured on pelvic X‐rays using the lateral centre edge angle (LCEA), ranges between 25° and 35° [27]. This is a rather wide range of 10° that allows for a wide selection of target zones for the lateral edge of the acetabular sourcil in PAO surgery. Furthermore, there is no evidence that a higher correction provides any survival benefit of the hip after PAO [28]. It is well established that PAO influences hip flexion and internal rotation. As demonstrated by Steppacher et al. and Hayashi et al., both three‐dimensional (3D) simulation studies and clinical trials have shown that increasing the LCEA after PAO is associated with decreased hip flexion and internal rotation [13, 25]. Nevertheless, the direct correlation between the degree of LCEA correction achieved during PAO and the corresponding change in range of motion (ROM) has not yet been investigated.

We therefore aimed to quantify the influence of the degree of lateral correction on the ROM of the hip after PAO. It was hypothesised that the higher the LCEA, the lower the hip flexion arc.

## METHODS

Computed tomography (CT) scans were obtained from 50 hips with symptomatic hip dysplasia confirmed on conventional antero‐posterior (AP) pelvic X‐rays based on established radiographic measures [27].

Hips were included if the LCEA was less than 25° on AP radiographs. Hips with normal or excessive lateral acetabular coverage or true acetabular retroversion with an LCEA of ≥25° were not considered ineligible for inclusion, despite a potential indication for PAO surgery. Moreover, hips with a Crowe classification >1, osteoarthritis graded >1 according to Tönnis, a history of previous surgeries, or the presence of femoral head deformity (Perthes‐like deformity or an alpha angle >60°), were considered unsuitable for this study.

The CT scans of 50 pelvises were included, of which the demographic data are represented in Table 2. Simulation was performed only for the dysplastic hip, and PAO simulation was executed unilaterally in all 50 cases.

### Imaging and three‐dimensional PAO simulation

All CT scans were performed according to a standard protocol with the patient lying in a supine position. Two short spiral axial scans spanning the pelvis in total as well as the distal femur, in slices of 512 pixels and a thickness of 0.5 mm.

Bony segmentation of all CT scans was performed to allow for subsequent PAO simulation. All PAO cuts were simulated and validated by an experienced PAO surgeon using MyPlanner software as previously described [1] (Medacta International, Rancate, Switzerland).

Correction duplicates were simulated for each Pelvic CT scan with a target LCEA value of 25° and 35°, respectively. Acetabular version was not changed to avoid potential confounders not related to the primary research question. Femoral torsion was measured using the Murphy method on the standardised CT scans including the proximal and distal femur. The calculated values were incorporated into the simulation. All CT scans were provided as two‐dimensional anteroposterior projection allowing for depiction of the radiographic measures used for dysplasia on conventional X‐rays after correction for pelvic tilt. Two independent investigators additionally measured anterior and posterior wall indices, acetabular index and before and after correction.

Hip motion was simulated before and after both corrections to depict ROM. Endpoints were defined as a bony conflict. All degrees of motion including extension, flexion, abduction, adduction as well as internal rotation and external rotation were noted. Internal/external rotation of the hip was measured in both neutral position and in 90° of flexion, corresponding to standard clinical exams.

### Statistical analysis

Continuous data are presented as median and interquartile range (IQR: 25th–75th percentile), with minimum and maximum values indicated where appropriate. Comparison between means was performed using a paired *t* test. Paired Mann–Whitney *U* tests were used to compare the distributions of three subgroups. A Friedman test was used to assess whether there were statistically significant differences in ROM among the three measurements. Linear regression analysis was utilised to identify measures influencing ROM. A corresponding adjustment was performed (Table 1). A post hoc power calculation confirmed that the study was well‐powered to detect the observed effect of LCEA correction on hip flexion and internal rotation (power > 0.99).

**Table 1:** Applied statistical tests and associated datasets.

Statistical method	Variable	Data set
Paired *t* test	Comparison between means (demographics, ROM)	(Figure 1, Tables 2 and 3)
Paired Mann–Whitney *U* test	Comparison between means of three subgroups (ROM)	Figure 2a,b
Friedman test	Assessment of differences among three measurements (ROM)	(Table 3)
Linear regression analysis	Identification of measures independently influencing ROM	(Table 4/Table 5)

Abbreviation: ROM, range of motion.

## RESULTS

The mean age of the cohort was 28.34 ± 10.38 years. Of the 50 patients included in this study, 5 (10%) were male and 45 (90%) female. Following PAO, all radiographic parameters related to femoral head containment demonstrated significant change (Table 2).

**Table 2:** Descriptive data of the study cohort.

Number of patients	Cohort	*p* value
Ø Age (years)	28.34 ± 10.38	
Sex		
Male	5/50 (10%)	<0.001
Radiographic data	45/50 (90%)	
Preoperative LCEA (°)	14.73 ± 5.89	
Preoperative AI (°)	14.93 ± 5.73	
Postoperative AI (LCEA 25°) (°)	4.12 ± 5.14	<0.001
Postoperative AI (LCEA 35°) (°)	−5.69 ± 4.69	<0.001
Preoperative anterior head coverage (%)	22.01 ± 3.49	
Postoperative anterior head coverage (LCEA 25°) (%)	24.35 ± 3.29	<0.001
Postoperative anterior head coverage (LCEA 35°) (%)	26.42 ± 4.6	0.003
Preoperative posterior head coverage (%)	31.45 ± 3.88	
Postoperative posterior head coverage (LCEA 25°) (%)	34.41 ± 1.97	<0.001
Postoperative posterior head coverage (LCEA 35°) (%)	36.94 ± 2.49	<0.001
Femoral torsion (°)	24.21 ± 13.25	

Abbreviations: AI, acetabular index; LCEA, lateral centre edge angle.

**Figure 1:**
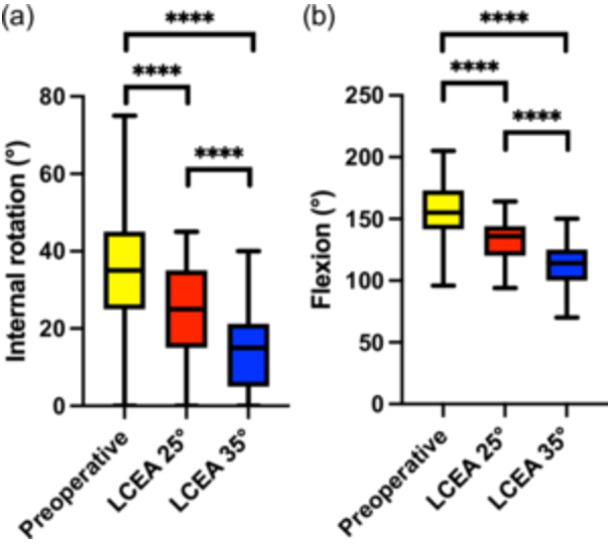
(a) Internal rotation in 90° flexion preoperatively and after simulated correction to lateral centre edge angle (LCEA) 25° and LCEA 35°. A significant reduction in internal rotation was observed with increasing lateral coverage (*p* < 0.0001) for all comparisons. (b) Progressive decline in hip flexion was significantly associated with greater LCEA correction (*p* <  0.0001).

Simulation of acetabular reorientation resulted in a significant reduction in hip internal rotation across all degrees of correction. Mean internal rotation decreased progressively from the preoperative condition to the simulated postoperative states at both LCEA 25° and LCEA 35°. This reduction was highly significant across all comparisons (*p* < 0.0001) (Figure 1a and Table 3).

**Table 3:** Change in postoperative range of motion in relation to LCEA correction.

Movement	Preoperative	LCEA 25°	LCEA 35°	*p* value
Extension	86.44 ± 71.15	86.84 ± 71.15	70.02 ± 34.36	0.628
Flexion	159.76 ± 40.64	132.45 ± 18.35	112.43 ± 18.55	<0.001
Internal rotation	35.5 ± 16.04	25.0 ± 10.88	14.2 ± 10.37	<0.001
External rotation	118.6 ± 27.55	118.9 ± 32.69	110.1 ± 46.19	0.005
Abduktion	77.0 ± 41.37	73.5 ± 25.27	73.5 ± 25.27	<0.001
Adduktion	62.48 ± 9.92	60.7 ± 20.76	63.16 ± 10.29	0.891

Abbreviation: LCEA, lateral centre edge angle.

Similarly, hip flexion demonstrated a statistically significant decrease from the preoperative state to both levels of LCEA correction, with all comparisons showing significance (*p* < 0.0001) (Figure 1b and Table 3). Whereas abduction and extension showed no significant differences when comparing preoperative to postoperative ROM as well as in relation to the LCEA correction, adduction and external rotation were significantly reduced postoperatively and depending on the extent of LCEA correction (Table 3).

**Figure 2:**
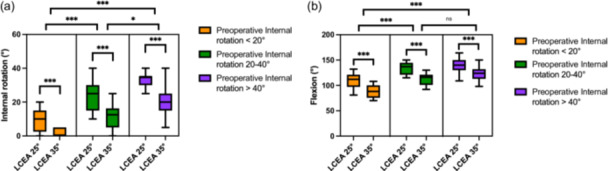
(a) The simulated internal rotation (°) in 90° flexion after correction to lateral centre‐edge angle (LCEA) 25° and 35°, stratified by preoperative internal rotation. Patients with low preoperative internal rotation ( < 20°) experienced the most pronounced reduction in internal rotation at both correction levels. All subgroup comparisons were statistically significant; however, the degree of significance differed. The reduction in internal rotation was highly significant between patients with low and moderate preoperative rotation (*p* < 0.001), while the difference between the moderate and high rotation groups reached statistical significance to a lesser extent (*p* =  0.024). (b) The simulated hip flexion after correction to LCEA 25° and 35°, stratified by preoperative internal rotation. Hip flexion decreased significantly across all subgroups with increasing correction. A nonsignificant difference was noted between the moderate and high internal rotation groups (*p* = 0.816); all other comparisons were highly significant (*p* < 0.001).

Subgroup analysis revealed that preoperative internal rotation significantly influenced the degree of motion loss following PAO. Patients with low preoperative internal rotation ( <20°) demonstrated the most pronounced reduction in internal rotation at both LCEA 25° and 35° compared with those with moderate (20°–40°) and high ( >40°) preoperative rotation (Figure 2a).

Similarly, hip flexion decreased significantly in all subgroups after correction to a LCEA of 25° and 35°. The loss of flexion was most evident in patients with limited preoperative hip flexion, with statistically significant differences observed between all subgroups (*p* < 0.001), except between the moderate and high internal rotation groups, where no significant difference was detected (Figure 2b).

**Figure 3:**
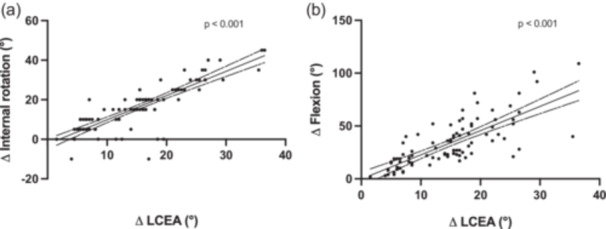
Scatter plots illustrating the relationship between changes in lateral centre‐edge angle (ΔLCEA) and changes in hip range of motion: (a) internal rotation and (b) flexion. Each dot represents an individual simulation of a specific ΔLCEA value and its corresponding degree of hip motion. A significant negative correlation was observed in both plots (*p* < 0.001). The solid line indicates the line of best fit, while the dashed lines represent the 95% confidence interval for the regression line.

**Figure 4:**
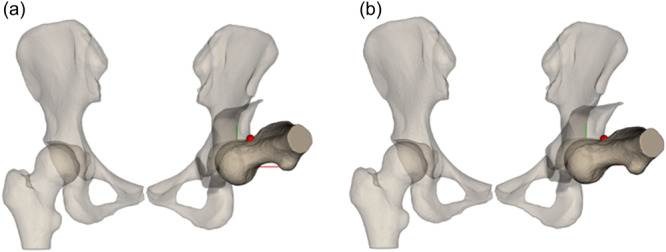
(a) Depiction of the conflict zone after a correction of with a target lateral centre‐edge angle (LCEA) of 25°. Here internal rotation with the hip in 90° of flexion was 35°. (b) Depiction of the conflict zone after a correction of with a target LCEA of 35° in the same hip. Here, internal rotation with the hip in 90° of flexion was 25°.

A statistically significant relationship was observed between changes in LCEA and hip ROM following PAO surgery. As shown in Figure 3a, increases in LCEA were significantly associated with decreases in internal rotation, with a clear negative correlation (*p* <0.001). In regard to hip flexion, Figure 4b demonstrated a significant inverse relationship between LCEA correction and changes in hip flexion (*p* < 0.001).

Multivariate linear regression analysis revealed that an increase of LCEA was significantly associated with a postoperative loss of internal rotation. Specifically, for each degree of LCEA, internal rotation decreased by 1.003° (*p* = 0.016, *R*² = 0.876). Moreover, preoperative internal rotation also was a strong predictor of postoperative internal rotation (*p* < 0.001) (Table 4).

**Table 4:** Multivariable linear regression analysis for independent factors that influence the degree of postoperative internal rotation.

Dependent factor	Independent factor			Confidence interval		
Postoperative Internal rotation (°)		Beta (β)	Regression coefficient (B)	Lower Limit	Upper Limit	Sig.
	Preoperative Internal rotation (°)	0.875	0.875	0.792	0.958	<0.001
	Δ LCEA (°)	−1.003	−1.003	−1.278	−0.729	0.016
	Femoral anteversion (°)	0.049	−0.049	−0.116	0.017	0.114
	Preoperative anterior wall index (%)	0.298	0.298	−0.094	0.689	0.134

Abbreviation: LCEA, lateral centre‐edge angle.

Similarly, flexion range was significantly impacted by the change of LCEA. Each degree of LCEA correction resulted in a loss of 1.36° of hip flexion (*p* < 0.001, *R*² = 0.763). Preoperative degree of flexion had also a significant influence (*p* < 0.001) on the level of postoperative flexion (Table 5).

**Table 5:** Multivariable linear regression analysis for independent factors that influence the degree of postoperative hip flexion.

Dependent factor	Independent factor			95% Confidence interval		
Postoperative Flexion (°)		Beta (β)	Regression coefficient (B)	Lower Limit	Upper Limit	Sig.
	Preoperative flexion (°)	0.833	0.606	0.514	0.697	<0.001
	Δ LCEA (°)	−0.5	−1.363	−2.022	−0.705	<0.001
	Femoral anteversion (°)	0.068	0.107	−0.061	0.275	0.211
	Preoperative anterior wall index (%)	0.046	0.28	‐0.722	1.283	0.58

Abbreviation: LCEA, lateral centre edge angle.

## DISCUSSION

The principal finding of this study is the quantifiable relationship between increased lateral coverage in PAO surgery and subsequent reduction in hip ROM. Specifically, for each degree of increase in lateral coverage, flexion decreases by 1.36° and internal rotation by 1.003°.

This observation underscores the critical importance of establishing individualised target values for correction in PAO surgery. Considering the wide range of normal lateral coverage, typically defined by a LCEA of 25°–35°, pursuing a higher correction target of 35° could result in a >10° additional loss of ROM in both flexion and internal rotation. Such a reduction may be sufficient to precipitate clinically relevant impingement, particularly in patients presenting with reduced preoperative internal rotation (Figure 4).

The results clearly demonstrate that preoperative internal rotation significantly influences postoperative internal rotation outcomes. High preoperative internal rotation appears to be protective, as any reduction resulting from PAO is unlikely to shift the arc of motion into a conflict zone.

However, particular caution is warranted in cases presenting with reduced preoperative internal rotation. Our findings indicate that hips with less than 20° of internal rotation preoperatively are at high risk of severely compromised internal rotation capability postoperatively, especially when corrected to a LCEA of 35°.

This sheds light at the problem of post‐PAO hip impingement that has been outlined in the literature [21, 29]. However, most of the literature referred to the problem of poor orientation and retroversion of the fragment, or the presence of a CAM deformity [3].

A study that was performed using pre‐ and postoperative CT scans in a series of 27 dysplastic hips undergoing PAO by the Boston group examined the change in ROM after PAO and highlighted the increase in prevalence of extra‐articular impingement on the anterior‐inferior iliac spine after PAO surgery [25]. A further study looked into the CT scans before and after curved osteotomy and emphasised the effect of PAO on internal rotation of the hip based on clinical evaluation of ROM [12]. In a subsequent study, the same authors highlighted the detrimental effects of overcorrection on ROM [13].

The current study explored this question in a controlled manner via duplicate simulations and confirmed the findings in a controlled experimental setting and demonstrated the outcome using a linear regression model. The findings of the current study underline the fact that even in a well‐performed PAO, the higher the degree of correction, the lower the internal rotation.

Analysis of our data suggests that hips with preoperative internal rotation of less than 20° are at significant risk of substantial ROM loss following PAO. This risk is particularly pronounced in cases where a larger correction is planned, targeting a LCEA of 35°. In such instances, these hips are likely to experience a complete loss of internal rotation and may be limited to 90° of flexion postoperatively. These findings underscore the importance of careful preoperative assessment and surgical planning, especially in patients with limited preoperative internal rotation. A more conservative target correction, aiming for a LCEA of 25°, may be protective and favourable in these cases.

Femoral torsion is also a well‐recognised determinant of hip kinematics and impingement, and clinical studies have shown a strong correlation between torsion and passive hip rotation [6]. In our analysis, however, linear regression including femoral torsion as a variable identified LCEA coverage as the primary factor influencing postoperative ROM. From a clinical perspective, it is important to note that surgical correction of femoral torsion, typically by intertrochanteric osteotomy, represents a substantially more invasive procedure than adjusting LCEA correction and may even result in nonphysiological hip joint mechanics [23]. Therefore, femoral torsion should be evaluated in every case of DDH; however, correction should be reserved for severe mal‐torsion, while balanced acetabular reorientation appears most appropriate for optimising hip motion after PAO.

In regard to clinical outcome, several studies highlight the clinical importance of avoiding excessive acetabular correction. Fan et al. reported that a postoperative LCEA greater than 38° was significantly associated with worse patient‐reported outcomes after PAO [8]. Similarly, Hayashi et al. demonstrated that excessive anterior coverage or high combined coverage and femoral version values predispose to postoperative femoroacetabular impingement and impaired clinical function [12, 13, 14]. These findings emphasise that while sufficient lateral coverage is essential for joint stability, overcorrection of the LCEA may diminish hip ROM and compromise patient outcomes, underlining the need for careful preoperative planning and balanced correction. Several biomechanical studies have highlighted that, in addition to potential restrictions in hip motion, increased acetabular coverage after PAO create also important biomechanical changes. Interestingly, Knight et al. demonstrated that PAO can increase cartilage stresses, decrease contact area and reduce joint congruency [16]. In addition, Goetz et al. reported that elevated contact stresses and smaller contact areas may persist after PAO [10]. Thus, although PAO remains a powerful tool to prevent or delay osteoarthritis, current literature suggests that increasing the lateral coverage of the femoral head only moderately alters joint loading forces and labral loading. There is therefore a lack of clear evidence in the literature supporting a slight overcorrection to higher LCEA values on the basis of a marked improvement in joint biomechanics.

The study has several limitations, primarily its reliance on CT‐based analysis, which exclusively assesses bony conflicts. This approach may potentially deviate from real‐case scenarios where soft tissue interactions also play a role. Nevertheless, the controlled nature of the study allows for precise delineation of bony effects.

In our PAO simulation, we deliberately maintained anterior and posterior wall coverage within a 2% range of change to minimise confounding factors. This constraint, while enhancing the study's focus, does not fully capture the complexity of actual surgical outcomes. Importantly, the role of acetabular version in PAO surgery is of paramount significance and merits further investigation. Our study's limited exploration of this factor underscores the need for additional research to comprehensively understand its impact on surgical outcomes and postoperative hip biomechanics.

To conclude, this study demonstrates that in PAO surgery, for every degree of increase in lateral coverage, flexion and internal rotation are reduced by 1.36° and 1.003°, respectively. Hips with preoperative internal rotation of <20° are at high risk of shifting the ROM arc into a conflict zone, especially if the LCEA is corrected to 35°. Accepting a correction on the lower end of the normal range, with a target LCEA of 25°, allows for significant maintenance of residual ROM after PAO.

## AUTHOR CONTRIBUTIONS

**Sufian S. Ahmad**: Formal analysis; funding acquisition; supervision; writing–original draft; investigation. **Justus Stamp**: Formal analysis; funding acquisition; supervision; writing–original draft; investigation. **Gaia Guidici**: Data curation; writing–review & editing; methodology. **Quentin Karisch**: Formal analysis; writing–review & editing. **Henning Windhagen**: Funding acquisition; writing–review & editing. **Marco Haertlé**: Data curation; formal analysis; funding acquisition; visualisation; writing–original draft. Sufian S. Ahmad and Justus Stamp contributed equally to this work and share first authorship.

## CONFLICT OF INTEREST STATEMENT

The authors’ research group receives annual funding by the non‐profit Erwin‐Röver‐Foundation, Germany, and Hannover Medical School, Germany. In addition, Sufian S. Ahmad, Gaia Guidici, and Henning Windhagen report grants or contracts and consulting fees from Medacta International, while Sufian S. Ahmad has consulting fees from Medacta International. Henning Windhagen also discloses consulting fees Aesculap/B.Braun; payment or honoraria for lectures, presentations, speakers bureaus, manuscript writing or educational events from Aesculap/B.Braun, Medacta International, and Stryker; and a leadership or fiduciary role for the Personalised Arthroplasty Society and Springer.

## ETHICS STATEMENT

The study was conducted in accordance with the Declaration of Helsinki and approved by the local Ethics Committee of the Hannover Medical School (code: 12011‐BO‐K‐2025).

## Data Availability

The data that support the findings for this study are available to other researchers from the corresponding author upon reasonable request.
